# Graphene mobility mapping

**DOI:** 10.1038/srep12305

**Published:** 2015-07-24

**Authors:** Jonas D. Buron, Filippo Pizzocchero, Peter U. Jepsen, Dirch H. Petersen, José M. Caridad, Bjarke S. Jessen, Timothy J. Booth, Peter Bøggild

**Affiliations:** 1 DTU Nanotech - Department of Micro- and Nanotechnology, Technical University of Denmark, Building 345 Ørsteds Plads, 2800 Kgs. Lyngby, Denmark; 2 DTU Fotonik - Department of Photonics Engineering, Technical University of Denmark, Building 343 Ørsteds Plads, 2800 Kgs. Lyngby, Denmark; 3 DTU Center for Nanostructured Graphene (CNG), DTU Nanotech - Department of Micro- and Nanotechnology, Technical University of Denmark, Building 345 Ørsteds Plads, 2800 Kgs. Lyngby, Denmark

## Abstract

Carrier mobility and chemical doping level are essential figures of merit for graphene, and large-scale characterization of these properties and their uniformity is a prerequisite for commercialization of graphene for electronics and electrodes. However, existing mapping techniques cannot directly assess these vital parameters in a non-destructive way. By deconvoluting carrier mobility and density from non-contact terahertz spectroscopic measurements of conductance in graphene samples with terahertz-transparent backgates, we are able to present maps of the spatial variation of both quantities over large areas. The demonstrated non-contact approach provides a drastically more efficient alternative to measurements in contacted devices, with potential for aggressive scaling towards wafers/minute. The observed linear relation between conductance and carrier density in chemical vapour deposition graphene indicates dominance by charged scatterers. Unexpectedly, significant variations in mobility rather than doping are the cause of large conductance inhomogeneities, highlighting the importance of statistical approaches when assessing large-area graphene transport properties.

Graphene^1^ is being targeted for an increasing number of commercially oriented applications^2^,^3^,^4^,^5^,^6^,^7^,^8^,^9^ and industrial development^10^,^11^,^12^, merited by its combination of exceptional electronic, optical, and mechanical properties as well as the increasing availability of synthesized large-area graphene films. In particular, there is a profound interest in the commercial adaptation of large-area graphene of high electrical quality for electronics applications, including high frequency terahertz (THz) electronics^13^,^14^,^15^ and transparent, flexible, and durable electrodes for graphene-based touch-screens, displays and solar cells^14^. There have been several demonstrations of THz time-domain spectroscopy (THz-TDS) for rapid and contact-free conductance measurements of large-area graphene^16^,^17^,^18^, making practical implementations of in-line spatial mapping of graphene sheet conductance on a large scale possible^19^,^20^,^21^. For typical electronic applications^22^, however, the conductance of a graphene film in itself gives an incomplete picture in terms of the expected performance. For many scientific as well as commercial applications, the carrier mobility and the background carrier density originating from intentional^23^,^24^ or unintentional^25^,^26^ chemical doping are the essential figures of merit^27^,^28^, and the most straightforward way to obtain this information is through the electric field effect, which requires a variable gate potential. Such characterization in contacted graphene devices ultimately results in the dissection and processing of the sample, if any kind of spatial or statistical information is required. This is a destructive process, where the question as to whether the final devices truly represent the initial state of the unprocessed film of graphene, is difficult to answer.

Here we demonstrate quantitative mapping of the field-effect carrier mobility in a large-area monolayer CVD graphene film based on *in-situ* electrically gated THz-TDS imaging. In contrast to prior electrically gated THz spectroscopy experiments performed on graphene^29^,^30^,^31^,^32^ we employ a low-mobility, high carrier concentration gate-electrode material, in this case p^+^ boron-doped, nano-crystalline silicon, to ensure a negligible THz response from free carriers injected into the gate-electrode. This allows for isolation of the graphene sheet conductance from non-contact THz-TDS transmission measurements and thus a quantitative extraction of the graphene field-effect mobility.

## Results

### Experiment and substrate design

To avoid THz absorption from free carriers in the substrate, to obtain a voltage-independent gate capacitance and to ensure an insignificant gate-differential THz response from free carriers injected into the gate-electrode, we employ a low-mobility, high carrier density thin film gate-electrode. This allows for isolation of the gate-induced conductance of the graphene and a quantitative extraction of the graphene field-effect mobility. To this end, we use a substrate comprised of 525 μm ρ  > 10,000 Ωcm high resistivity silicon (Si), 50 nm boron-doped poly-crystalline Si (poly-Si), and 165 nm Si nitride (Si_3_N_4_). The THz response of the poly-Si/Si_3_N_4_ stack is found to be negligible with a frequency-independent transmission of 100% ± 1% and a diminishing phase-shift of 2 ° (see Supplementary information), which is attributed to slight differences in high resistivity Si substrate thickness between the positions of the reference and sample measurements. In accordance, the 2-point contact DC sheet conductance of the poly-Si thin film was measured to be below σ_*s,poly-Si*_ = 0.1 mS, which is at the limit of sensitivity of the THz spectrometer. Combined with a Boron doping concentration of *n*_*poly-Si *_= 2.6 × 10^19 ^cm^−3^, measured by secondary ion mass spectroscopy, the sheet conductance value is consistent with an expected carrier mobility below *μ*_*poly-Si *_= 10 cm^2^/Vs.^33^,^34^,^35^

**Figure 1:**
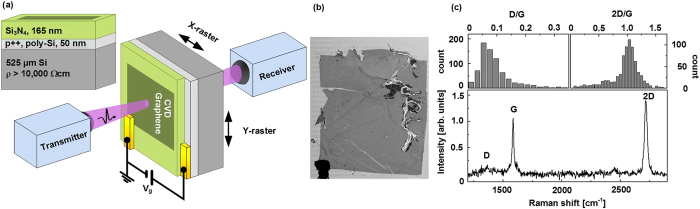
(**a**) Schematic of sample consisting of large-area monolayer CVD graphene film residing on a layered substrate comprising 525 μm high resistivity Si, 50 nm boron-doped poly- Si with n ≈ 2.6 × 10^19 ^cm^−3^, and 165 nm Si_3_N_4_. Graphite and gold contacts are used for contacting the graphene film and the poly-crystalline Si film, respectively. (**b**) tiled optical ultra-high resolution image of CVD graphene sample on THz-transparent, layered substrate. (**c**) Raman characteristics of CVD graphene film, including representative spectrum, D/G and 2D/G distributions

A large-area graphene film is grown on commercially available Cu foil by means of a standard catalytic chemical vapour deposition (CVD) and is subsequently transferred onto the layered substrate, as shown in Fig. 1(a), by a standard polymer-assisted technique, involving complete sacrificial etching of the Cu substrate in (NH_4_)_2_S_2_O_8_. Figure 1(b) and (c) shows a tiled, high-resolution optical image and Raman analysis characteristics of the final sample, respectively.

### Back-gated terahertz conductance mapping

**Figure 2:**
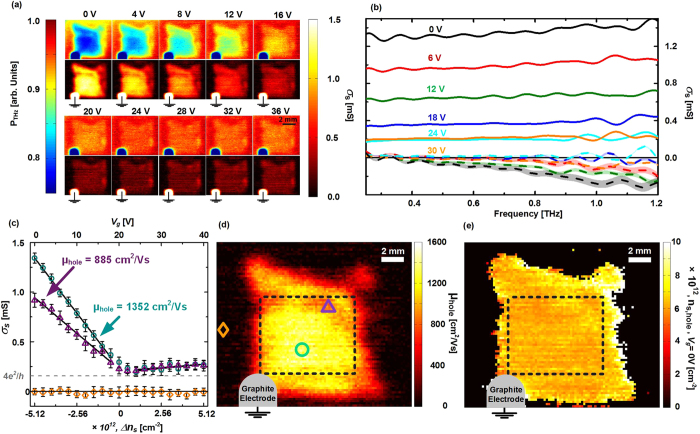
(**a**) Raster images obtained at different gate voltages showing the transmitted THz power as well as average, real sheet conductance value from 0.5–0.9 THz. Scale bars are 5 mm. See Supplementary information for overlays with the optical image in Fig. 1(b) (**b**) Graphene sheet conductance spectra from the central region at series of different gate voltages. Full lines: real part. Dashed lines: imaginary part. Uncertainty on Re[*σ*_s_] and Im[*σ*_s_] due to substrate thickness variations of 200 nm shown as shaded confidence bands (**c**) Average, real, gate-induced sheet conductance from 0.5 to 0.9 THz as a function of *V*_*g*_ for 3 distinct positions of the mapped area. Circles, triangles and squares are experimental data and the full lines are linear fits to the data for 0 V < *V*_*g *_< 18 V and 26 V < *V*_*g *_< 40 V for hole and electron mobilities, respectively. (**d**)+(**e**) maps showing the spatial distribution of hole field effect mobility, *μ*_*FE*,_ and carrier density, n_s,_ at *V*_*g *_= 0 V across the CVD graphene film evaluated at 0.5–0.9 THz.

THz maps of the CVD graphene on Si_3_N_4_/poly-Si/Si were recorded at 21 different gate voltages between 0V and 40V, a selection of which are shown in Fig. 2(a), where the data is represented by images showing the total transmitted THz power and corresponding THz sheet conductance. Since the CVD graphene film is heavily p-doped, the conductance and therefore the THz response of the graphene film are lowest in the *V*_*g *_=24 V THz map. The complex, frequency-dependent sheet conductance 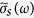 can be directly obtained in each pixel of the maps from the measured amplitude and phase change upon transmission through the sample (see Methods section). As shown in Fig. 2(b) the THz sheet conductance exhibits a real part of the conductance which rises very slightly with increasing frequency, and exhibits a strong dependence on the applied gate voltage. The imaginary part of the conductance, which is subject to a relatively high uncertainty due to substrate thickness variations on the order of 200 nm across the 2 cm sample (see Supplementary information), is close to 0 in the accessible frequency range of 0.25–1.2 THz, and it exhibits a weak dependence on both frequency and applied gate voltage. The slightly rising real conductance along with an imaginary conductance which decreases slightly towards more negative values with increasing frequency is consistent with the Drude-Smith model, and may suggest a small degree of preferential back-scattering of charge carriers in this particular graphene film, as we recently reported on^36^. For frequencies significantly below the scattering rate (*2πf≪1/τ*), the Drude-Smith and Drude models, expected for the THz response of pristine graphene, dictates that the real part of 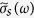 is constant and near its DC value while the imaginary part is close to zero. Our spectrally resolved measurement can thus be replaced by a single, real-valued quantity reflecting the DC conductance of the graphene film.

In Fig. 2(a) we have used this approach to form maps of the graphene sheet conductance, represented by the average value of 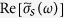 between 0.5–0.9 THz, from here on referred to as *σ*_*s*_. The evolution of *σ*_*s*_ with varying *V*_*g*_ in the series of images in Fig. 2(a) is a result of the electric field effect in graphene and thus provides vital information on the carrier mobility and chemical doping of the film.

### Field-effect mobility mapping

Figure 2(c) shows σ_*s*_ as a function of *V*_*g*_ for three distinct areas in the map with highly conducting graphene, less conducting graphene, and no graphene coverage. The measured of σ_*s*_ shows a linear dependence on the applied gate voltage in the range 0V < *V*_*g *_< 18 V with different slopes throughout the extent of the graphene film, reflecting the local field-effect mobility for hole carriers. This observation is found to be representative throughout the graphene film area. For gate voltages 24 V < *V*_*g *_< 40 V a plateau with slowly increasing σ_*s*_ is found. This observation indicates that electron-conduction is greatly impaired in this particular graphene film with electron mobilities that are more than an order of magnitude lower than the observed hole mobilities. Application of a gate voltage, V_g_, between the poly-Si thin film and the graphene film induces a proportional change in the free carrier density, *Δn*_*s*_, in the poly-Si and graphene films, as 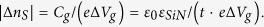 Throughout the extent of the film, a highly consistent minimum is found in the sheet conductance close to *V*_*g *_= 24 V. This minimum represents the charge-neutrality-point (CNP), indicating a constant, and homogeneous presence of residual absorbates with a density of approximately 5 × 10^12 ^cm^−2^, which p-dopes the entire graphene film. Within the regime of long-range, charged impurity scattering, the added free graphene carriers result in a roughly linear change in the graphene sheet conductance, *σ*_*s*_, with the field effect mobility being a proportionality factor. The sheet conductance, *σ*_*s*_, of the graphene film is thus described by the relations^37^:


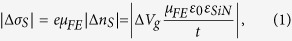



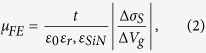


where *C*_*g*_ is the gate capacitance, *e* is the electronic charge, ε_*0*_ is the vacuum permittivity, ε_*SiN *_= 7.5 is the relative permittivity of Si nitride, *t* is the thickness of Si nitride gate dielectric, and *μ*_*FE*_ is the field-effect mobility of the graphene film.

The gate-induced conductance in the gate electrode can be neglected because of the insignificant carrier mobility (*μ*_*poly-Si *_< 10 cm^2^/Vs) of our poly-Si thin film relative to the hole mobility of the investigated graphene film, which is approximately two orders of magnitude higher. Because of the choice of a low-mobility, high carrier concentration gate material, the experiments presented here provide a direct and reliable measure of the gate-induced conductance evolution of the graphene film with an insignificant contribution from gate-induced carriers in the substrate. Following equation (2), the field effect mobility of the graphene film can thus be obtained in each pixel of the mapped area by retrieving the slope *|Δ*σ_*s*_*/ΔV*_*g*_| in the high density-region well below the CNP. Based on this central result we are able to show a spatially resolved field-effect hole mobility map for the large-area CVD graphene film in Fig. 2(d), resulting from the application of equation (2) to the entire dataset. The slope |*Δ*σ_*s*_*/ΔV*_*g*_| was found in each pixel by a linear fitting routine to σ_*s*_ vs. *V*_*g*_ from 0 V to +18 V. Similarly, the carrier density due to chemical doping of the graphene film at zero gate bias, can be evaluated across the map from the determined hole mobilities and the sheet conductance at Vg = 0, by utilizing the relation σ_*s*_ *= en*_*s*_*μ*_*hole*_. Figure 2(e) shows a map of *n*_*s*_ (*n*_*s*_ is set to 0 in pixels where *μ*_*hole *_< 100 cm^2^/Vs). We note that carrier mobility measured using this technique is affected by the coverage of graphene, because equations (3) and (4) assume full areal coverage within the measurements area. In case of less than full coverage, mobilities are underestimated by a factor proportional to the convolution of the THz spot profile and the local graphene coverage landscape.

## Discussion

In contrast to recent back-gated THz spectroscopy investigations of CVD graphene^29^,^31^, our measurement of σ_*s*_ shows a linear dependence on the applied gate voltage, reflecting the local field-effect mobility for hole carriers. The linear dependence of *σ*_*s*_ on *V*_*g*_ is expected for graphene films where long-range, charged impurity scattering dominates over short-range neutral defect scattering^37^,^38^, and is typically observed in DC transport measurements of samples with field effect mobility *μ *< 5.000 cm^2^/Vs.^1^,^23^,^39^,^40^ Our findings of a spatially homogeneous doping level of approximately 5 × 10^12^ cm^−2^, as well as strongly suppressed electron mobility are consistent with transport measurements on graphene in atmospheric conditions. It is well known that a presence of O_2_ and H_2_O adsorbates on graphene causes a strong p-doping on the monolayer, which is particularly relevant in samples not annealed and exposed to atmospheric conditions^40^,^41^, as is our case. In addition, this unintentional p-doping inhibits the mobility of electrons relative to that of holes. The latter effect was predicted as a unique property of massless fermions^42^,^43^, and has been verified several times in conventional DC transport measurements^23^,^40^. The relatively high chemical doping density further supports the notion that the electronic transport is dominated by long-range scattering on charged impurities^37^.

**Figure 3:**
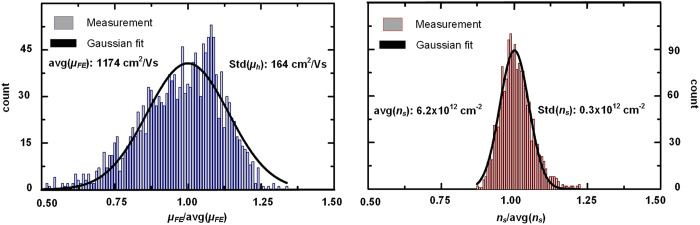
Histograms with measured *μ*_*hole*_ and *n*_*s,hole*_ within the dashed, black rectangles Fig. 2 in (d) and (e).

Unexpectedly, Fig. 2 (d) and (e) reveal that the large-scale spatial variations in the sheet conductance of the graphene film, observed in Fig. 2(a), are primarily due to spatial variations in the carrier mobility, rather than variations in doping level. The chemical doping level, shown in Fig. 2(e) presents only small spatial fluctuations. In contrast, we find that even on a scale of a few mm, the carrier mobility varies by up to a factor 2, highlighting the importance of techniques that either facilitate a statistical approach for assessing transport properties in CVD graphene or allow spatial mapping of graphene transport properties such as that presented here. This fact is quantified in Fig. 3, which shows histograms of *μ*_*hole*_ and *n*_*s*_(0V) from the central region of the graphene film, marked by dashed, black rectangles in Fig. 2(d) and (e). The fitted Gaussian distributions plotted along with the distributions, have peak values of *μ*_*hole *_= 1174 cm^2^/Vs and *n*_*s*_(0 V) = 6.2 × 10^12 ^cm^−2^, and relative standard deviations of std(*μ*_*hole*_)/avg(*μ*_*hole*_) = 14.0% and std(*n*_*s*_)*/*avg(*n*_*s*_) = 4.8%.

We find that the procedure presented here gives far more comprehensive information than the typical convention of relying on a few representative electrical measurements from random or selected areas of a large graphene film. We also note that the features and inhomogeneities in the field effect mobility in Fig. 2(d) were not observed in a typical Raman analysis of the amplitude-ratio of the the D- and G-bands, which can be found in the Supplementary information.

We anticipate that quantitative, non-contact mapping of graphene carrier mobility as presented in this work will have a significant technological importance for the advancement of production and implementations of large-area graphene in commercial electronic applications and that it can contribute to the fundamental insight into charge carrier dynamics of the graphene solid-state-system by paving the way for investigations of transport dynamics with unprecedented statistical basis.

## Conclusions

In conclusion, we have presented non-contact, quantitative mapping of graphene field effect mobility in a 10 × 10 mm^2^ large-area CVD film by *in-situ* electrically gated THz-TDS measurements. While THz-TDS is non-contact, optical characterization, the frequency range is well below the inverse scattering time of the graphene charge carriers, making the obtained conductance directly comparable the DC value. The technique thus opens up the possibility for assessment of fundamental electrical transport properties such as carrier mobility and chemical doping level on basis of large statistical ensembles, or large-area spatial mapping. Far from the charge-neutrality-point, our measurements show a linear dependence of the THz sheet conductance on carrier density, which is interpreted as a signature of electrical transport limited by long-range, charged impurity scattering, also observed in most DC transport measurements on graphene. Unexpectedly, we find that the mobility varies by up to a factor of 2 on a scale of just few mm, and that significant conductance variations in the investigated CVD graphene are due to mobility rather doping variations, which highlights the importance of techniques that facilitate highly statistical or spatially resolved approaches for assessing transport properties in large-area graphene.

## Methods

### Terahertz time-domain spectroscopy

The experiments are carried out using a Picometrix^©^ T-ray™ 4000 fiber-coupled THz time-domain spectrometer relying on photoconductive switches for THz generation and detection, which is detailed elsewhere^21^. The sample is placed in the THz focal plane formed between 2 aspheric polymer lenses, each with a working distance of 25.4 mm, producing a focused spot size of approx. 0.6 mm at 0.5 THz and 0.3 mm at 0.9 THz^21^. By raster-scanning the graphene sample in steps of 0.2 mm in the THz focus, pulse waveforms are recorded in every pixel of a spatially resolved map with close to 7500 pixels covering the full extent of the 10 × 10 mm^2^ graphene film. Through utilization of the electric field effect in graphene, THz maps are recorded at different carrier densities by applying voltages, *V*_*g*_, in the range from 0 V to 40 V between poly-Si thin film and graphene film. The full acquisition time for each map is 16 minutes, and *V*_*g*_ is increased in discrete steps of 2 V between each map.

The sheet conductance of the graphene film was extracted in each pixel of the THz maps based on an approach described previously^21^. The complex-valued transmission coefficient, 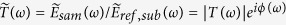 in each pixel of the map can be directly related to the complex sheet conductance, 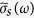, of the graphene film according to equation , which is derived from Fresnel coefficients for the boundaries in the sample geometry, modeled as an infinitely thin conducting surface of sheet conductance, 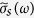
44,45 on a thick high resistivity Si dielectric medium with refractive index n_Si _= 3.417^46^. 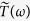 is calculated as the ratio of the Fourier transform of the transmitted sample THz waveform, 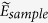, (transmission through graphene/Si_3_N_4_/poly-Si/Si) to that of a reference waveform, 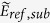
(transmission through Si_3_N_4_/poly-Si/Si-substrate), and can be related to real and imaginary parts of the sheet conductance as


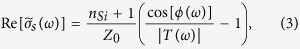



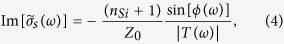


where Z_0 _= 377 Ω is the vacuum impedance.

## Additional Information

**How to cite this article**: Buron, J. D. *et al.* Graphene mobility mapping. *Sci. Rep.* **5**, 12305; doi: 10.1038/srep12305 (2015).

## Supplementary Material

Supplementary Information
