# Supplementary material for: Graphene mobility mapping
Source: Sci Rep. 2015 Jul 24;5:12305. doi: 10.1038/srep12305 (PMC4513276; doi:10.1038/srep12305)
Supplement: Supplementary Information [file srep12305-s1.pdf]

Supplementary information: Graphene mobility Mapping

Jonas D. Buron[1], Filippo Pizzocchero[1], Peter U. Jepsen[2], Dirch H. Petersen[1], José M. Caridad, Bjarke S. Jessen, Timothy J. Booth, and Peter Bøggild[1,3,*]

[1]*DTU Nanotech - Department of Micro- and Nanotechnology, Technical University of Denmark, Building 345 Ørsteds Plads, 2800 Kgs. Lyngby, Denmark*
[2]*DTU Fotonik - Department of Photonics Engineering, Technical University of Denmark, Building 343 Ørsteds Plads, 2800 Kgs. Lyngby, Denmark*
[3]*DTU Center for Nanostructured Graphene (CNG), DTU Nanotech - Department of Micro- and Nanotechnology, Technical University of Denmark, Building 345 Ørsteds Plads, 2800 Kgs. Lyngby, Denmark*

THz response of poly-Si/Si_3N_4 stack

The THz transmission characteristics of the layered poly-Si/Si_3N_4 structure are measured by transmission THz-TDS. Sup. fig. 1(a) shows THz time-domain transients transmitted through the bare high resistivity silicon substrate and the layered high resistivity silicon/poly-Si/Si_3N_4 structure, respectively, and sup. fig. 1(b) shows the corresponding frequency-dependent amplitude transmission coefficient, $T_{poly\text{-}Si/SiN}$, and phase-shift, $\phi_{poly\text{-}Si/SiN}$, associated with transmission through the poly-Si/Si_3N_4 stack, which are related to the Fourier transforms of the data in sup. fig. 1(a) as

$$\frac{\tilde{E}_{HR-Si/poly-Si/SiN}(\omega)}{\tilde{E}_{HR-Si}(\omega)} = T_{poly-Si/SiN}\, e^{i\phi_{poly-Si/SiN}(\omega)} \tag{1}$$

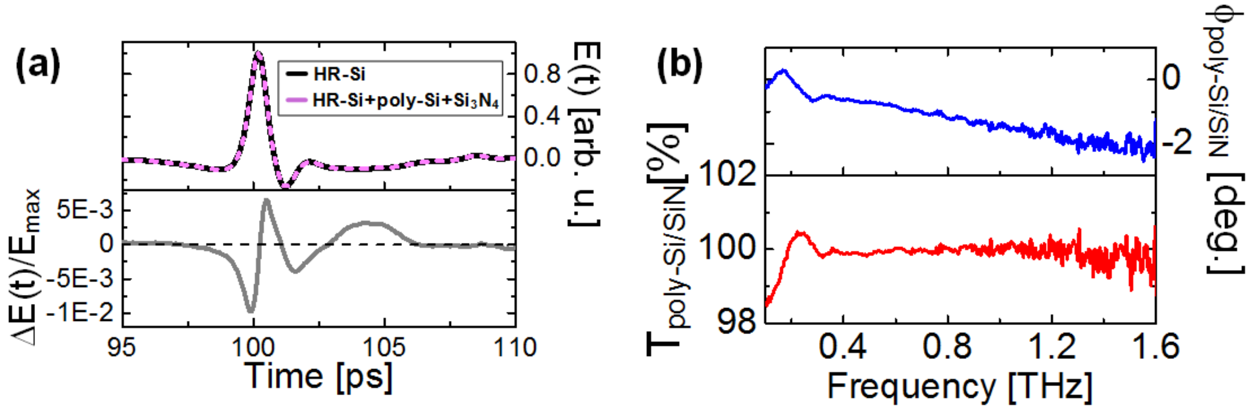

Sup. Fig. 1: (a) Time-domain waveforms of THz pulses transmitted through a bare HR-Si substrate and a layered HR-Si/poly-Si/Si_3N_4 substrate **(b)** Frequency-dependent amplitude transmission, $T_{poly\text{-}Si/SiN}$, and phase-shift, $\phi_{poly\text{-}Si/SiN}$, for poly-Si and Si_3N_4 layers. The THz response of the thin films is found to be negligible.

Raman D/G analysis

The Raman response of the CVD graphene film was mapped using a ThermoFischer DXR µ-Raman microscope. The excitation wavelength was 445 nm, the laser spot size was approx. 1µm^2, and the excitation power was 2 mW. Below is a map showing the amplitude ratio of the D and G bands, as well as 2D and G bands, the position and width of the 2D band, and the position and width of the G band. The amplitude, position, and widths of the Raman bands were obtained by fitting a single Gaussian function to each of the D, G, and 2D bands separately.

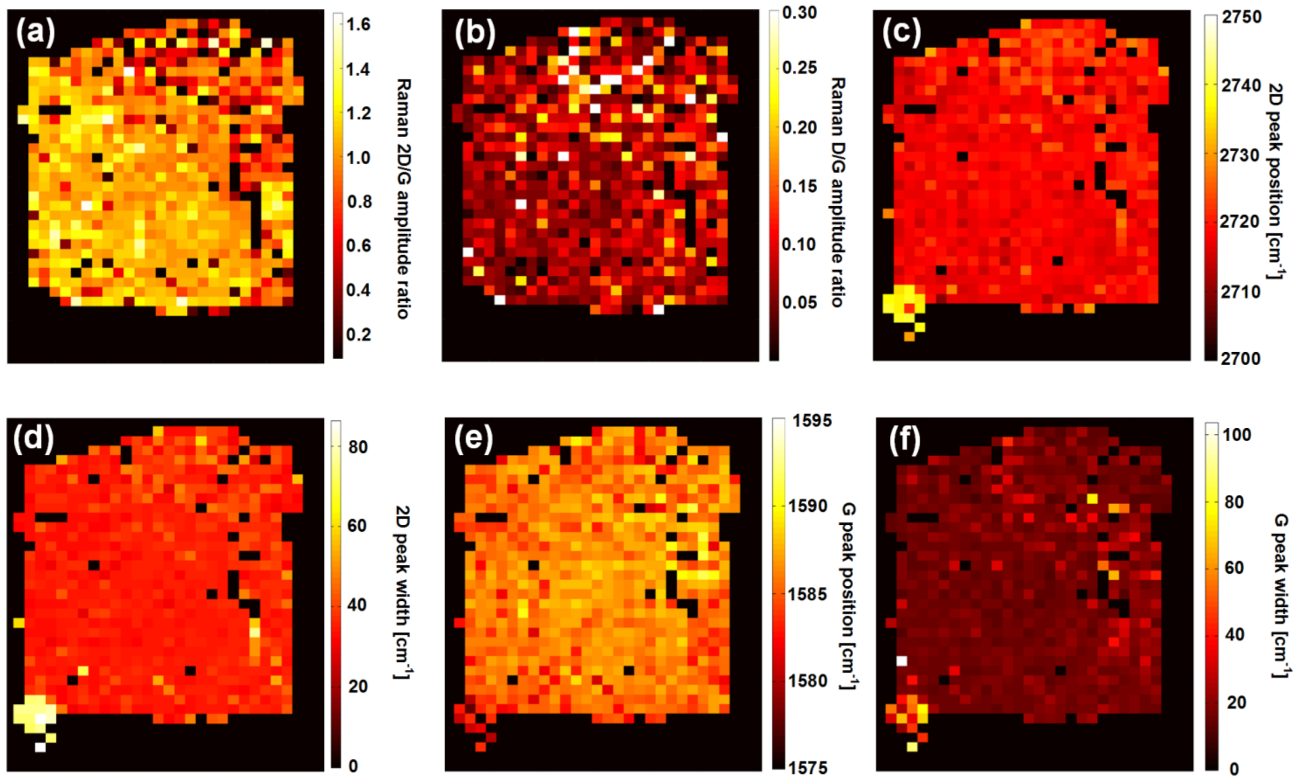

Sup. Fig. 2: µ-Raman maps of (a) amplitude ratio of 2D/G bands, (b) amplitude ratio of D/G bands, (c) 2D band peak position, (d) 2D band peak width, (e) G band peak position, and (f) G band peak width

Evaluating the error on extracted THz conductivity due to substrate thickness gradients

Since the substrates used for this study are state-of-the-art, double-side-polished silicon wafers, spatial thickness gradients across the substrate can be expected to be on the sub-micron scale. The contribution to the extracted THz conductivity from the added phase resulting from these variations will be considered in the following.

Because of the way the data is analyzed, the extracted THz conductance is only affected by the thickness difference, Δd, in the "horizontal" direction of our presented THz images. For each "horizontal" line of the raster-scanned images, a reference waveform is defined as the average of the 5 rightmost pixels, which is used to evaluate the THz sheet conductance in each pixel of that same line. (see sup. fig. 3 for sketch of the scheme)

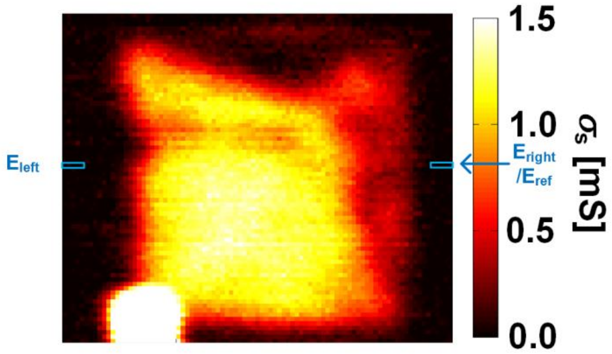

Sup. Fig. 3

To evaluate the magnitude of thickness gradients in the horizontal direction across the sample, we examine the phase difference between the THz waveforms transmitted in the left and right sides of the substrate. To this end, we evaluate ϕ_{sub} for each horizontal line, according to

$$\tilde{T}(\omega) = \frac{\tilde{E}_{left}(\omega)}{\tilde{E}_{right}(\omega)} = |T(\omega)| e^{i\phi_{sub}}, \tag{2}$$

where $\tilde{E}_{left}(\omega)$ and $\tilde{E}_{right}(\omega)$ are the average Fourier transforms of the THz waveforms recorded in the 5 leftmost and 5 rightmost pixels, respectively. The average value of ϕ_{sub} is plotted in the black curve in sup. fig. 4 as a function of frequency, with the standard deviation of ϕ_{sub} plotted as the shaded band.

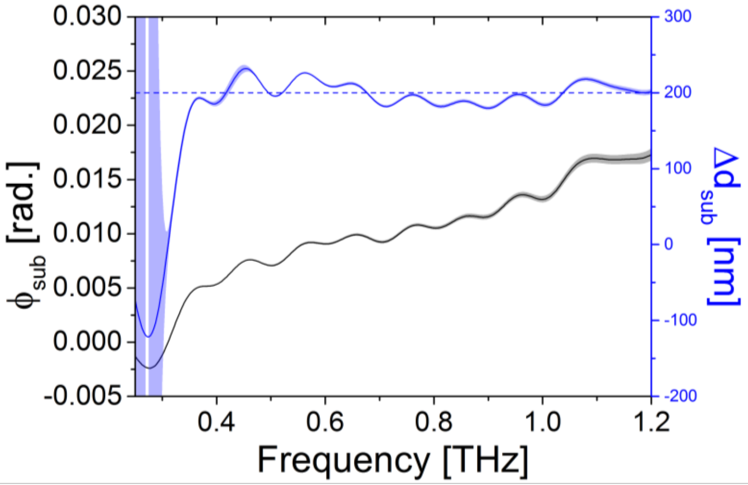

Sup. Fig. 4

For non-absorbing high-resistivity silicon, ϕ_{sub} can be related to a substrate thickness difference, Δd_{sub}, between left and right sides of the sample by

$$\Delta d_{sub} = \frac{\phi_{sub} c}{\omega n_{Si}}, \tag{3}$$

which is plotted in the blue curve in fig. sup. 4. This analysis shows that the substrate thickness difference from left to right side of the sample is approximately 200 nm.

As the real and imaginary conductances are given as

$$\mathrm{Re}\left[\tilde{\sigma}_s\right] = \frac{n_{Si}+1}{Z_0}\left(\frac{\cos(\phi)}{\left|\tilde{T}(\omega)\right|}-1\right), \tag{4}$$

$$\mathrm{Im}\left[\tilde{\sigma}_s\right] = -\frac{(n_{Si}+1)}{Z_0}\frac{\sin(\phi)}{\left|\tilde{T}(\omega)\right|}, \tag{5}$$

the error on these quantities due to the phase error of a 200 nm substrate thickness variation, Δd_{sub}, can be evaluated by propagation of Gaussian errors as

$$S_{\mathrm{Re}[\sigma_s]} = \left|\frac{\partial(\mathrm{Re}[\sigma_s])}{\partial\phi}\right| S_\phi = \sin(\phi)\frac{n_{Si}+1}{Z_0\left|\tilde{T}(\omega)\right|}S_\phi = \sin(\phi)\frac{n_{Si}+1}{Z_0\left|\tilde{T}(\omega)\right|}\frac{\omega n_{Si}\Delta d_{sub}}{c} \tag{6}$$

$$S_{\mathrm{Im}[\sigma_s]} = \left|\frac{\partial(\mathrm{Im}[\sigma_s])}{\partial\phi}\right| S_\phi = \cos(\phi)\frac{n_{Si}+1}{Z_0\left|\tilde{T}(\omega)\right|}S_\phi = \cos(\phi)\frac{n_{Si}+1}{Z_0\left|\tilde{T}(\omega)\right|}\frac{\omega n_{Si}\Delta d_{sub}}{c}. \tag{7}$$

For the small phase values ($\phi << 1$) obtained in graphene films, these expressions can be linearized as

$$S_{\mathrm{Re}[\sigma_s]} = \sin(\phi)\frac{n_{Si}+1}{Z_0\left|\tilde{T}(\omega)\right|}S_\phi \approx \phi\frac{n_{Si}+1}{Z_0\left|\tilde{T}(\omega)\right|}\frac{\omega n_{Si}\Delta d_{sub}}{c} \tag{8}$$

$$S_{\mathrm{Im}[\sigma_s]} = \cos(\phi)\frac{n_{Si}+1}{Z_0\left|\tilde{T}(\omega)\right|}S_\phi \approx \left(1-\phi^2\right)\frac{n_{Si}+1}{Z_0\left|\tilde{T}(\omega)\right|}S_\phi \approx \frac{n_{Si}+1}{Z_0\left|\tilde{T}(\omega)\right|}\frac{\omega n_{Si}\Delta d_{sub}}{c}. \tag{9}$$

For small phase values ($\phi << 1$), we can thus expect the impact on the real conductivity to be much smaller than the impact on the imaginary conductivity, since

$$\frac{S_{\mathrm{Im}[\sigma_s]}}{S_{\mathrm{Re}[\sigma_s]}} = \frac{1}{\phi} \tag{10}$$

Using the found Δd_{sub} = 200 nm substrate thickness difference as an uncertainty on the substrate thickness, the uncertainty in $\mathrm{Re}\left[\tilde{\sigma}_s\right]$ and $\mathrm{Im}\left[\tilde{\sigma}_s\right]$ can be evaluated and plotted with the data, as shown in sup. fig. 5.

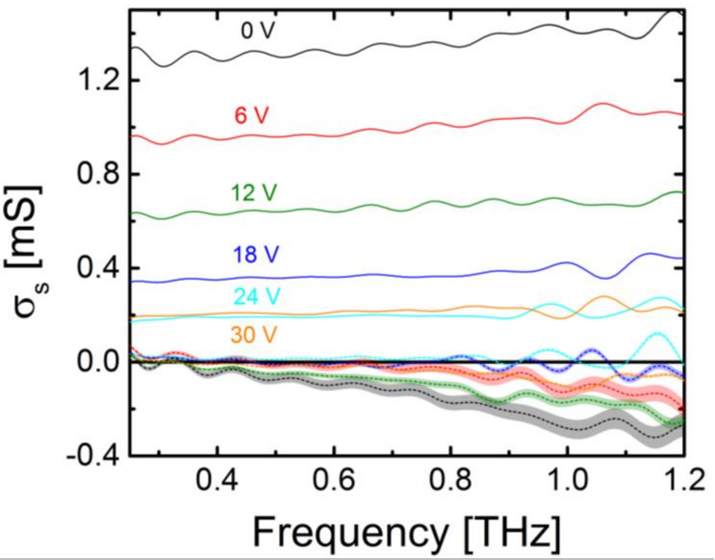

Sup. Fig. 5

The influence on the real part of the extracted sheet conductance is negligible, as expected, while the impact on the imaginary part of the extracted sheet conductance, on the other hand, is significant, and linearly increasing with frequency.

Optical-THz overlay

To provide some clarity on the spatial correlations between the optical features and electrical sheet conductance features of the investigated graphene film, sup. fig. 6 shows an overlay of σ_s@0Vg on a tiled optical micrograph, for varying values of the transparency of the conductance overlay map.

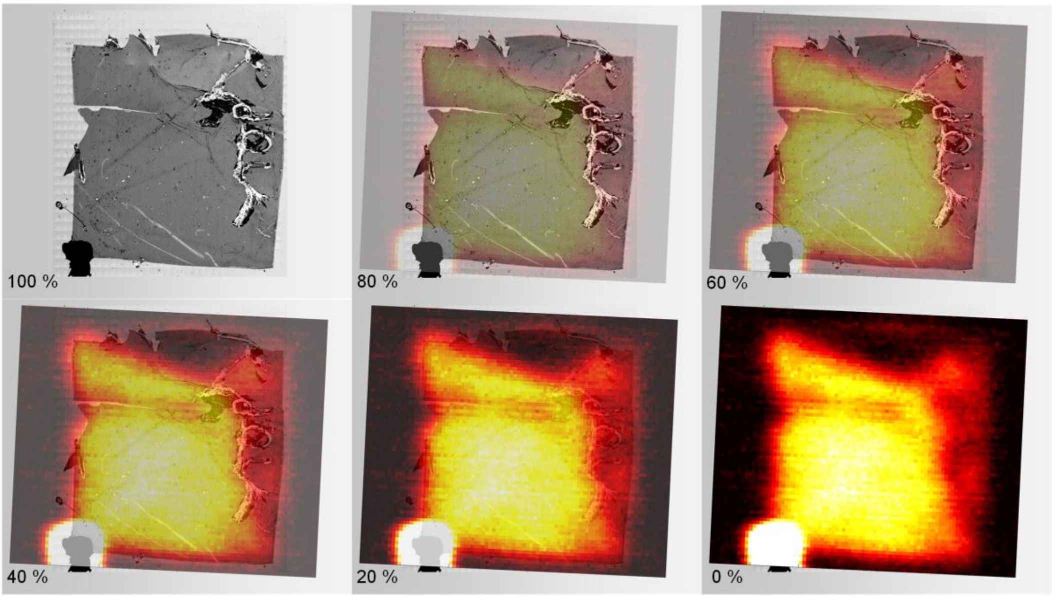

Sup. Fig. 6 – The transparency of the THz conductance overlay map is indicated in percent.
